# Extended prophylaxis for venous thromboembolism after hospitalization for medical illness: A trial sequential and cumulative meta-analysis

**DOI:** 10.1371/journal.pmed.1002797

**Published:** 2019-04-29

**Authors:** Navkaranbir S. Bajaj, Muthiah Vaduganathan, Arman Qamar, Kartik Gupta, Ankur Gupta, Harsh Golwala, Javed Butler, Samuel Z. Goldhaber, Mandeep R. Mehra

**Affiliations:** 1 Division of Cardiology, Department of Internal Medicine and Radiology, University of Alabama at Birmingham, Birmingham, Alabama, United States of America; 2 Brigham and Women’s Hospital Heart and Vascular Center and Harvard Medical School, Boston, Massachusetts, United States of America; 3 Department of Internal Medicine, All India Institute of Medical Sciences, New Delhi, India; 4 Division of Cardiology, Department of Internal Medicine, UTSW Medical Center, Dallas, Texas, United States of America; 5 Department of Medicine, University of Mississippi, Jackson, Mississippi, United States of America; Leiden University Medical Center, NETHERLANDS

## Abstract

**Background:**

The efficacy, safety, and clinical importance of extended-duration thromboprophylaxis (EDT) for prevention of venous thromboembolism (VTE) in medical patients remain unclear. We compared the efficacy and safety of EDT in patients hospitalized for medical illness.

**Methods and findings:**

Electronic databases of PubMed/MEDLINE, EMBASE, Cochrane Central, and ClinicalTrials.gov were searched from inception to March 21, 2019. We included randomized clinical trials (RCTs) reporting use of EDT for prevention of VTE. We performed trial sequential and cumulative meta-analyses to evaluate EDT effects on the primary efficacy endpoint of symptomatic VTE or VTE-related death, International Society on Thrombosis and Haemostasis (ISTH) major or fatal bleeding, and all-cause mortality. The pooled number needed to treat (NNT) to prevent one symptomatic or fatal VTE event and the number needed to harm (NNH) to cause one major or fatal bleeding event were calculated.

Across 5 RCTs with 40,247 patients (mean age: 67–77 years, proportion of women: 48%–54%, most common reason for admission: heart failure), the duration of EDT ranged from 24–47 days. EDT reduced symptomatic VTE or VTE-related death compared with standard of care (0.8% versus 1.2%; risk ratio [RR]: 0.61, 95% confidence interval [CI]: 0.44–0.83; *p* = 0.002). EDT increased risk of ISTH major or fatal bleeding (0.6% versus 0.3%; RR: 2.04, 95% CI: 1.42–2.91; *p* < 0.001) in both meta-analyses and trial sequential analyses. Pooled NNT to prevent one symptomatic VTE or VTE-related death was 250 (95% CI: 167–500), whereas NNH to cause one major or fatal bleeding event was 333 (95% CI: 200–1,000). Limitations of the study include variation in enrollment criteria, individual therapies, duration of EDT, and VTE detection protocols across included trials.

**Conclusions:**

In this systematic review and meta-analysis of 5 randomized trials, we observed that use of a post-hospital discharge EDT strategy for a 4-to-6-week period reduced symptomatic or fatal VTE events at the expense of increased risk of major or fatal bleeding. Further investigations are still required to define the risks and benefits in discrete medically ill cohorts, evaluate cost-effectiveness, and develop pathways for targeted implementation of this postdischarge EDT strategy.

**Trial registration:**

PROSPERO CRD42018109151.

## Abstract 2

A meta analysis and trial sequential on the effects of post-hospital discharge treatment for venous thromboembolism reveals a reduction in subsequent fatal events, Mandeep Mehra and colleagues show.

## Author summary

**Why was this study done?:**

- Current guidelines advocate for use of venous thromboembolism (VTE) prophylaxis among hospitalized patients with an acute medical illness until discharge. However, the risk of VTE persists and is cumulative in the postdischarge phase over the subsequent 4 to 6 weeks.
- Several randomized clinical trials have evaluated the therapeutic effects of extended-duration thromboprophylaxis (EDT) in attenuating the accumulated VTE risk. Although reduction in VTE was seen in these trials, none of them individually demonstrated superiority of EDT over standard of care.
- Our principal aim was to evaluate the aggregate efficacy of EDT on clinically relevant endpoints and to ascertain the robustness of efficacy signals balanced against the safety of the EDT strategy.

**What did the researchers do and find?:**

- We performed a systematic review, trial sequential analysis, and cumulative meta-analysis to identify all randomized clinical trials (RCTs) that assessed EDT in medically ill patients and evaluate the aggregate efficacy of EDT on clinically relevant endpoints. We assessed the robustness of efficacy signals balanced against the safety of the EDT strategy.
- We identified 5 RCTs that compared EDT with standard of care in medically ill patients requiring hospitalization, most commonly for heart failure.
- We observed that EDT reduced symptomatic VTE or VTE-related death compared with standard of care at the expense of an increased risk of major or fatal bleeding in both trial sequential and cumulative meta-analyses.

**What do these findings mean?:**

- A post-hospital discharge EDT strategy of anticoagulation for a 4–6 weeks period reduces symptomatic or fatal VTE events in patients hospitalized for acute medical illness at the expense of increased risk of major or fatal bleeding. Further investigations are required to define risks and benefits as well as cost-effectiveness within specific populations of medically ill patients.

## Introduction

Current guidelines advocate for the use of venous thromboembolism (VTE) prophylaxis in hospitalized patients with an acute medical illness until the time of discharge [1]. However, the risk of VTE persists and is cumulative in the postdischarge phase over the subsequent 4 to 6 weeks. Several randomized clinical trials (RCTs) have evaluated the therapeutic effects of extended-duration thromboprophylaxis (EDT) in attenuating this accumulated VTE risk [2–4]. None of these trials, which now include the large MARINER (Medically Ill Patient Assessment of Rivaroxaban versus Placebo in Reducing Post-Discharge Venous Thrombo-Embolism Risk) trial, has convincingly demonstrated the superiority of EDT [5].

Previous meta-analyses have shown that EDT is associated with a reduction in VTE risk, largely driven by a reduction in asymptomatic VTE events, a finding that is counterbalanced by an increased propensity for bleeding complications [6–8]. Prior meta-analyses [7] and RCTs [2–4,9] included asymptomatic deep vein thrombosis (DVT) in the postdischarge period to establish the effect size for benefit. However, the clinical relevance of this endpoint may be questioned since routine screening lower extremity venous ultrasound scans are not typically performed in the postdischarge phase unless a clinical reason ensues. Furthermore, the evolution and prognosis of such asymptomatic thrombotic events remain uncertain.

Trials that measure treatment effects can demonstrate exaggerated effect sizes early in the chain of evidence, a phenomenon referred to as the “proteus effect” [10,11] of sequential accrual of information. It is important that evidence accrued from a large trial like MARINER be examined in the context of sequential accumulation of data from the prior clinical trials [5]. Thus, our principal aim in this meta-analysis was to evaluate the aggregate efficacy of EDT on clinically relevant endpoints and to ascertain the robustness of efficacy signals balanced against the safety of the EDT strategy. To accomplish this, we employed trial sequential analysis techniques to improve precision of effect sizes over time because data have continued to accumulate in the field of EDT.

## Materials and methods

Because this was a systematic review and meta-analysis of published trial results, institutional review board and ethics committee approval was not required.

### Protocol and registration

Our review was registered with PROSPERO with the registration number CRD42018109151.

This study was conducted in accordance with Preferred Reporting Items for Systematic Reviews and Meta-Analyses (PRISMA) guidelines (S1 PRISMA checklist).

### Eligibility criteria

We included RCTs enrolling adult patients (>18 years of age) hospitalized for acute medical illness and compared EDT with standard of care. We did not have any language exclusions. The search strategy is detailed in the supporting information (S1 Search Strategy).

### Search strategy and information sources

**Fig 1:**
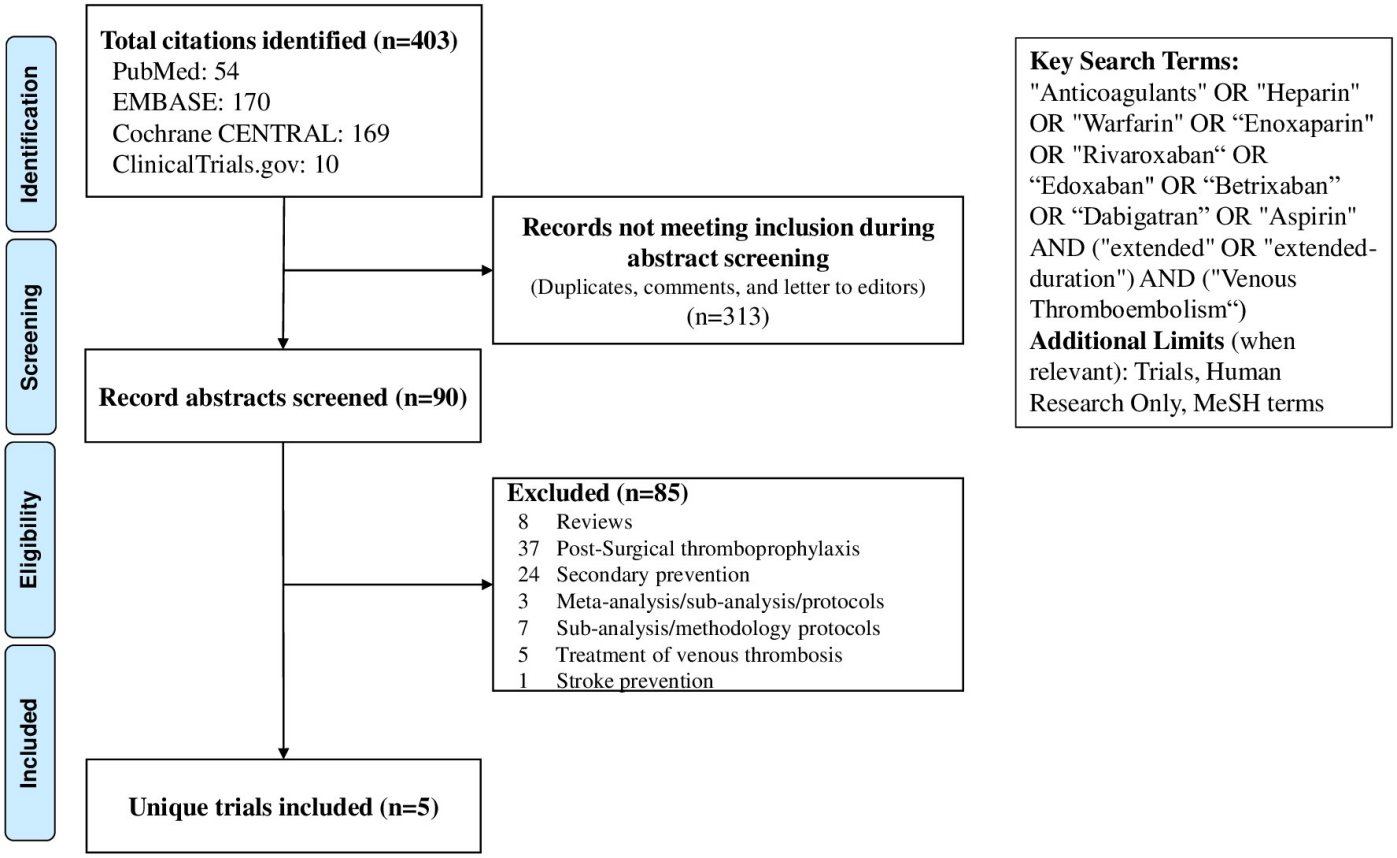
Flow diagram for study selection. MeSH, Medical Subject Headings.

A systematic PubMed/MEDLINE, EMBASE, Cochrane Central, and ClinicalTrials.gov search was performed from inception through March 21, 2019 using prospectively established criteria (Fig 1) for RCTs of EDT in hospitalized medically ill patients. Of 403 records screened by 3 independent investigators (AQ, KG, and AG), 5 RCTs were eligible for final inclusion. Titles and abstracts were screened initially, followed by full text retrieval of citations thought to be potentially eligible. Any disagreements were resolved by consensus or through discussion with the principal investigator (MRM).

### Data collection process

MV and AQ independently extracted data, and any inconsistencies were resolved by a third investigator (NSB).

### Data items

Data items extracted from each study included study characteristics, risk of bias (RoB) items, demographic information, treatment details, follow-up, and outcomes of interest.

### RoB in individual studies

RoB was assessed using the Cochrane RoB tool modified to capture the components of random sequence generation, allocation concealment, blinding of participants, blinding of outcome assessment, and analysis of incomplete outcome data.

### Outcomes

The principal efficacy endpoint examined in this analysis was symptomatic VTE or VTE-related death (S1 Table). We also extracted the primary efficacy endpoint selected by each trial, which was a composite of DVT, nonfatal pulmonary embolism, or VTE-related death. However, some trials excluded asymptomatic VTE or distal DVT (Table 1). To investigate the safety of EDT, we systematically evaluated the rates of major or fatal bleeding episodes as defined by the International Society on Thrombosis and Haemostasis (ISTH) criteria [12]. The definitions are detailed in S2 Table.

**Table 1:** Study designs, treatment protocols, and baseline patient profiles across the EDT trials.

Trial	MARINER [5]	APEX [4]	MAGELLAN [3]	ADOPT [2]	EXCLAIM [9]
**Study design**	Randomized, double blind, placebo-controlled, multicenter	Randomized double blind, double dummy, multicenter	Randomized double blind, double dummy, multicenter	Randomized double blind, double dummy, multicenter	Randomized double blind, multicenter
**Treatment arm**	Rivaroxaban 10 mg once daily†	Betrixaban 80 mg once daily	Rivaroxaban 10 mg once daily	Apixaban 2.5 mg twice daily	Enoxaparin 40 mg once daily
**Comparison**	EDT (rivaroxaban)	EDT (betrixaban)	EDT (rivaroxaban)	EDT (apixaban)	EDT (enoxaparin)
	SDT (placebo)	SDT (enoxaparin)	SDT (enoxaparin)	SDT (enoxaparin)	SDT (enoxaparin)
**Route of administration**	Oral	Oral	Oral	Oral	Subcutaneous
**Control arm**	Placebo	Enoxaparin for 10 ± 4 days followed by placebo	Enoxaparin for 10 ± 4 days followed by placebo	Enoxaparin for duration of hospital stay for a minimum of 6 days followed by placebo	Enoxaparin during hospitalization followed by placebo
**Duration of anticoagulation (days)**	45	35–42	35 ± 4	30	28 ± 4
**Primary efficacy outcome**	Symptomatic VTE or death related to VTE through day 45	Asymptomatic proximal DVT between days 32–47, symptomatic proximal or distal DVT, symptomatic nonfatal PE, or death related to VTE	Asymptomatic proximal DVT, symptomatic proximal or distal DVT, symptomatic nonfatal PE, or death related to VTE up to day 35	Asymptomatic proximal DVT, symptomatic proximal or distal DVT, symptomatic nonfatal PE, or death related to VTE	Symptomatic or asymptomatic proximal DVT, symptomatic PE, or fatal PE
**Primary safety outcome**	Major bleeding	Major bleeding at any point until 7 days after discontinuation of all study medications	Major bleeding or clinically relevant nonmajor bleeding observed no later than 2 days after discontinuation of all study medications	Major bleeding or clinically relevant nonmajor bleeding	Major bleeding during and up to 2 days after discontinuation of all study medications
**Number of patients randomized**	12,024	7,513	8,101	6,528	6,085
**Mean age, years**	69.7	76.6	71.0*	66.8	67.9
**Women, n (%)**	5,733 (47.7)	4,088 (54.4)	3,712 (45.8)	3,325 (50.9)	3,019 (49.6)
	**Reason for Hospitalization**				
**HF, n (%)**	4,835 (40.2)	3,349 (44.6)	2,620 (32.3)	2,516 (38.5)	1,110 (18.2)
**Acute ischemic stroke, n (%)**	1,726 (14.4)	843 (11.2)	1,399 (17.3)	NR	389 (6.4)
**Acute respiratory failure, n (%)**	3,186 (26.5)	922 (12.3)	2,268 (27.8)	2,421 (37.1)	1,805 (29.7)
**Acute inflammatory rheumatic diseases, n (%)**	175 (1.5)	226 (3.0)	303 (3.7)	124 (1.9)	173 (2.8)
**Active cancer, n (%)**	NR	NR	592 (7.3)	211 (3.2)	96 (1.6)
**Infection without septic shock, n (%)**	2,093 (17.4)	NR	3,682 (45.5)	1,447 (22.2)	1,982 (32.6)
**Other (plus not reported), n (%)**	NR	NR	58 (0.7)	20 (0.3)	408 (6.7)
	**Additional Risk Factors**				
**Age ≥75 years, n (%)**	4,294 (35.7)	5,092 (67.8)	3,116 (38.5)	NR	1,781 (29.3)
**Previous VTE, n (%)**	1,513 (12.6)	608 (8.1)	381 (4.7)	265 (4.1)	402 (6.6)
**History of HF (NYHA class III/IV), n (%)**	NR	1,718 (22.9)	2,790 (34.4)	2,478 (38.0)	1,110 (18.2)
**Acute infectious disease, n (%)**	NR	1,222 (16.3)	1,167 (14.4)	NR	NR
**History of cancer, n (%)**	1,021 (8.5)	909 (12.1)	1,378 (17.0)	632 (9.7)	817 (13.4)

* Median.

^†^7.5 mg once daily if CrCl 30–49 ml/min.

**Abbreviations**: ADOPT, Apixaban Dosing to Optimize Protection from Thrombosis; APEX, Acute Medically Ill Venous Prevention with Extended Duration Betrixaban; CrCl, creatinine clearance; DVT, deep vein thrombosis; EDT, extended-duration thromboprophylaxis; EXCLAIM, Extended Prophylaxis for Venous ThromboEmbolism in Acutely Ill Medical Patients With Prolonged Immobilization; HF, heart failure; MAGELLAN, Multicenter, Randomized, Parallel Group Efficacy and Safety Study for the Prevention of Venous Thromboembolism in Hospitalized Acutely Ill Medical Patients Comparing Rivaroxaban with Enoxaparin; MARINER, Medically Ill Patient Assessment of Rivaroxaban versus Placebo in Reducing Post-Discharge Venous Thrombo-Embolism Risk; NYHA, New York Heart Association; NR, not recorded; PE, pulmonary embolism; SD, standard deviation; SDT, standard-duration thromboprophylaxis; VTE, venous thromboembolism.

### Synthesis of results

#### Meta-analysis and publication bias

Random effects modeling was used to estimate summary risk ratios (RRs) for all outcomes. Data were analyzed for heterogeneity using the I^2^ statistic proposed by Higgins and Thompson; 95% confidence intervals (CIs) around I^2^ statistic were also estimated [13]. The Cochran’s Q, H-statistic, and Tau-squared using maximal likelihood and restricted maximal likelihood models were also estimated. A two-sided *p* < 0.05 was considered statistically significant. We intended to assess small study treatment effects using funnel plot techniques, Egger’s regression test, and Duval and Tweedie trim and fill methods as appropriate, given the known limitations of these methods [14,15]. Cumulative meta-analyses were performed in accordance with study by Lau and colleagues [16]. Trial sequential analysis was used to quantify the statistical reliability of data in cumulative meta-analyses by adjusting significance levels for sparse data and repetitive testing on accumulating data [17]. RoB for primary efficacy outcome was determined for each trial [18].

#### Trial sequential analysis

Most meta-analyses lack sufficient statistical power to detect treatment effects even when they are large [17]. When the number of included participants or trials is low, traditional meta-analytic techniques and statistical significance thresholds may lead to false positive (type I errors) or false negative conclusions (type II errors). In these situations, the Lan–DeMets trial sequential monitoring boundaries in trial sequential analysis offer adjusted CI when the required information size and the corresponding number of required trials for the meta-analysis have not been reached. Trial sequential analysis provides a frequentist approach to control both type I and type II errors. Several empirical studies have demonstrated that the trial sequential analysis provides better control of type I errors and of type II errors than traditional naïve meta-analysis [17,19]. A cumulative Z-curve was plotted against the accrued sample size. Lan–DeMets trial sequential boundary for benefit and harm were constructed, assuming the cumulative relative risk reduction for each outcome, α = 0.05, and β = 0.20.

We calculated the pooled number needed to treat (NNT) to prevent one symptomatic or fatal VTE event and the number needed to harm (NNH) to cause one major or fatal bleeding event. To estimate pooled NNT and NNH, random effects meta-analyses of risk difference were performed, and the pooled estimates derived from these analyses were inverted [20]. All analyses were performed using STATA V15.0 (College Station, TX, USA) statistical software.

#### Findings

The 5 RCTs [2–5,9] in this meta-analysis included 40,247 hospitalized medically ill patients. The duration of EDT ranged from 24–47 days, while the comparison control group typically used standard-duration thromboprophylaxis (range 6–14 days). Therapeutic regimens were a low-molecular weight heparin enoxaparin in one trial [9], while all others [2–5] investigated non-vitamin K antagonist oral anticoagulants that target inhibition of factor Xa. These included apixaban [2] (1 trial), betrixaban [4] (1 trial), and rivaroxaban [3,5] (2 trials). The mean/median age of trial participants in the RCTs varied from 66–71 years, with equitable gender distribution (women: 48%–54%). Heart failure was the most common medical illness requiring hospital admission (18%–45%) (Table 1). RoB was estimated using the RoB 2.0 tool and was deemed acceptable (Table 2).

**Table 2:** Revised tool to assess RoB in randomized trials (RoB 2.0).

Study Name	Year	Randomization Bias	Intervention Deviation	Missing Outcome Data	Measurement of Outcome	Reporting of Outcome	Overall Risk
**EXCLAIM**	2010	Low	Low	Some Concern	Low	Low	Low
**ADOPT**	2011	Low	Low	Some Concern	Low	Low	Low
**MAGELLAN**	2013	Low	Low	Some Concern	Low	Low	Low
**APEX**	2016	Low	Low	Some Concern	Low	Low	Low
**MARINER**	2018	Low	Low	Low	Low	Low	Low

**Abbreviations**: ADOPT, Apixaban Dosing to Optimize Protection from Thrombosis; APEX, Acute Medically Ill Venous Prevention with Extended Duration Betrixaban; EXCLAIM, Extended Prophylaxis for Venous ThromboEmbolism in Acutely Ill Medical Patients With Prolonged Immobilization; MAGELLAN, Multicenter, Randomized, Parallel Group Efficacy and Safety Study for the Prevention of Venous Thromboembolism in Hospitalized Acutely Ill Medical Patients Comparing Rivaroxaban with Enoxaparin; MARINER, Medically Ill Patient Assessment of Rivaroxaban versus Placebo in Reducing Post-Discharge Venous Thrombo-Embolism Risk; RoB, risk of bias.

#### Meta-analysis for primary efficacy endpoint

**Fig 2:**
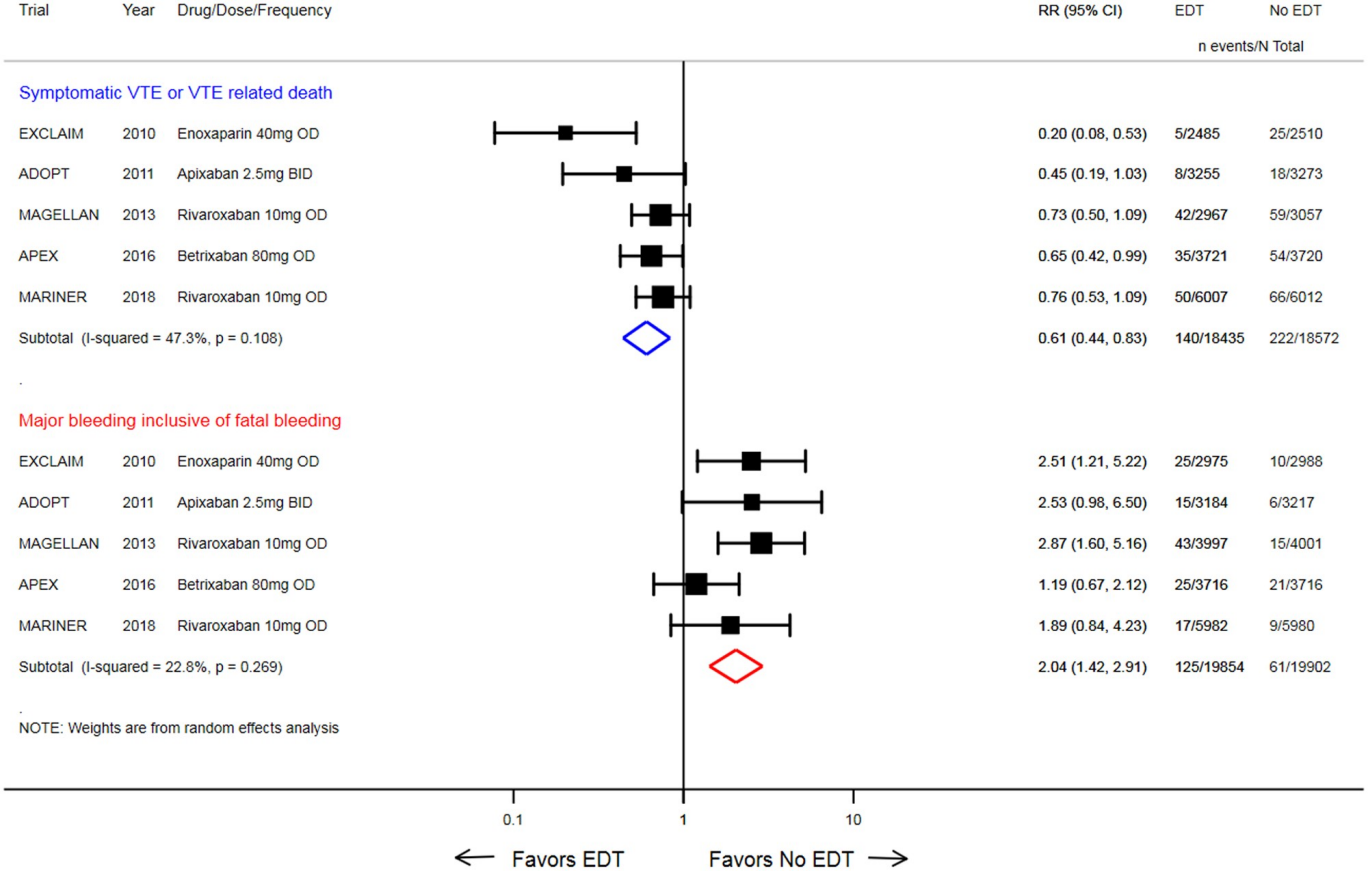
Forest plot comparing EDT versus standard-duration thromboprophylaxis in hospitalized medically ill patients for the primary efficacy endpoint (symptomatic VTE or VTE-related death) and the primary safety endpoint (major or fatal bleeding). Black solid square markers and associated solid lines represent summary RR and 95% CI of each trial listed in the left column. The size of black markers is proportional to standard error of effect estimate. The numerical estimates in the right columns are RRs with 95% CI of each trial listed in the left column. The hollow blue diamond is summary RR and 95% CI for VTE or VTE-related death, whereas the hollow red diamond is summary RR for major or fatal bleeding. ADOPT, Apixaban Dosing to Optimize Protection from Thrombosis; APEX, Acute Medically Ill Venous Prevention with Extended Duration Betrixaban; BID, two times a day; CI, confidence interval; EDT, extended-duration thromboprophylaxis; EXCLAIM, Extended Prophylaxis for Venous ThromboEmbolism in Acutely Ill Medical Patients With Prolonged Immobilization; MAGELLAN, Multicenter, Randomized, Parallel Group Efficacy and Safety Study for the Prevention of Venous Thromboembolism in Hospitalized Acutely Ill Medical Patients Comparing Rivaroxaban with Enoxaparin; MARINER, Medically Ill Patient Assessment of Rivaroxaban Versus Placebo in Reducing Post-Discharge Venous Thrombo-Embolism Risk; OD, once daily; RR, risk ratio; VTE, venous thromboembolism.

Traditional and cumulative meta-analysis showed that EDT significantly reduced symptomatic VTE or VTE-related death alone across the 5 trials when compared with standard of care (0.8% versus 1.2%; RR: 0.61, 95% CI: 0.44–0.83; *p* = 0.002) with moderate heterogeneity (I^2^ = 47.0%; *p* = 0.110 and Tau-squared = 0.0) (Fig 2). The heterogeneity for treatment effect of EDT on primary efficacy endpoint across trials was assessed using several methods and was deemed to be moderate (S3 Table). When the primary efficacy endpoints as selected by the individual trials were evaluated, EDT was associated with a 25% reduction in the risk of the trial-specified primary efficacy endpoint (2.8% versus 3.7%; RR: 0.75, 95% CI: 0.66–0.84; *p* < 0.001).

Small study treatment effects were not assessed because the number of included trials was inadequate to properly create a funnel plot or employ more advanced regression-based assessments.

#### Trial sequential analysis for primary efficacy endpoint

**Fig 3:**
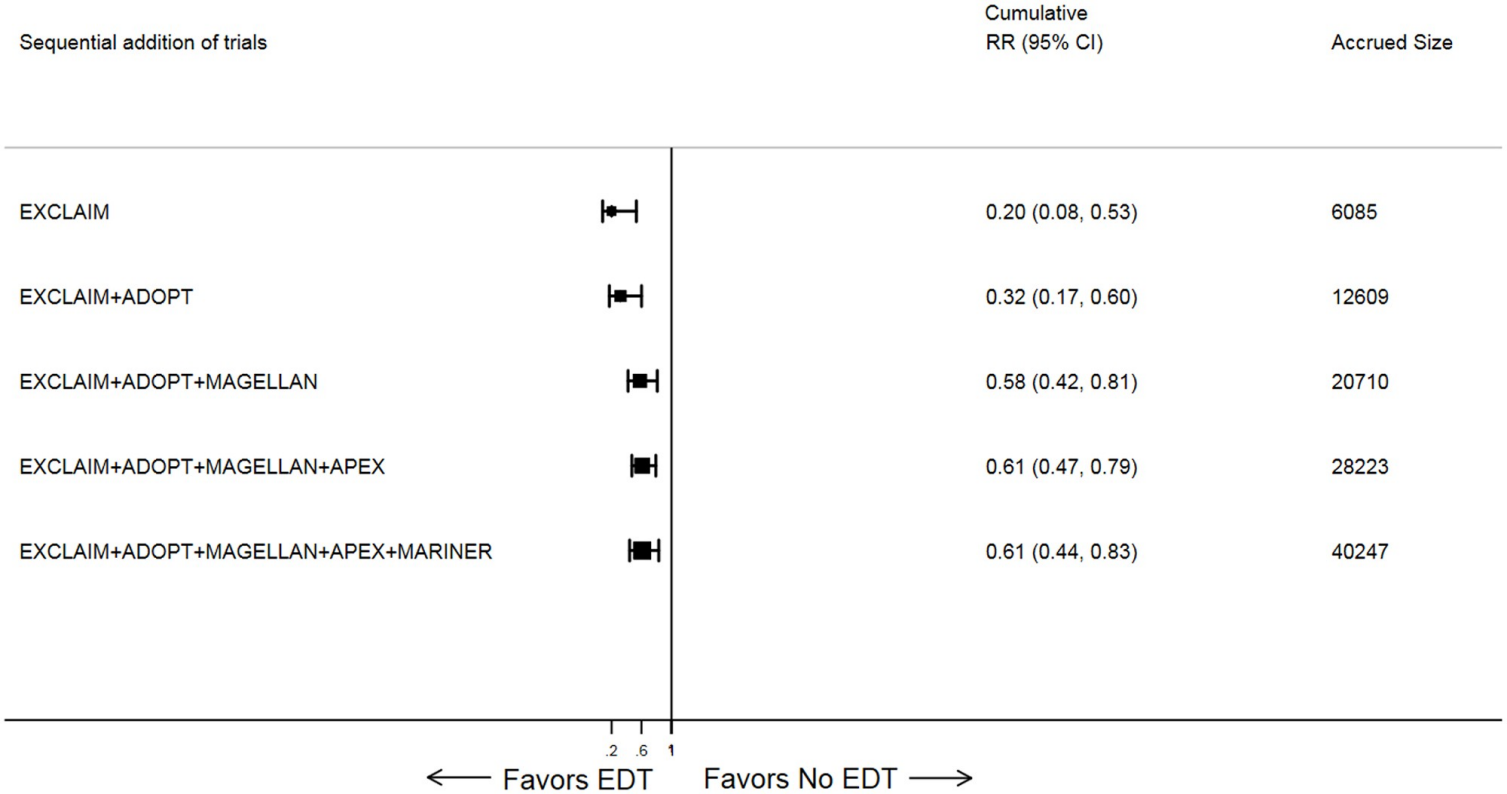
Forest plot for cumulative meta-analysis of EDT versus standard-duration thromboprophylaxis in hospitalized medically ill patients for primary efficacy events. Black solid square markers and associated solid lines represent cumulative summary RR and 95% CI after addition of each trial as listed in the left column. The size of black markers is proportional to total accrued size. The numerical estimates in the right columns are cumulative RRs and 95% CI after sequentially adding each trial and cumulative sample size after addition of each trial listed in left column. ADOPT, Apixaban Dosing to Optimize Protection from Thrombosis; APEX, Acute Medically Ill Venous Prevention with Extended Duration Betrixaban; CI, confidence interval; EDT, extended-duration thromboprophylaxis; EXCLAIM, Extended Prophylaxis for Venous ThromboEmbolism in Acutely Ill Medical Patients With Prolonged Immobilization; MAGELLAN, Multicenter, Randomized, Parallel Group Efficacy and Safety Study for the Prevention of Venous Thromboembolism in Hospitalized Acutely Ill Medical Patients Comparing Rivaroxaban with Enoxaparin; MARINER, Medically Ill Patient Assessment of Rivaroxaban versus Placebo in Reducing Post-Discharge Venous Thrombo-Embolism Risk; RR, risk ratio.

**Fig 4:**
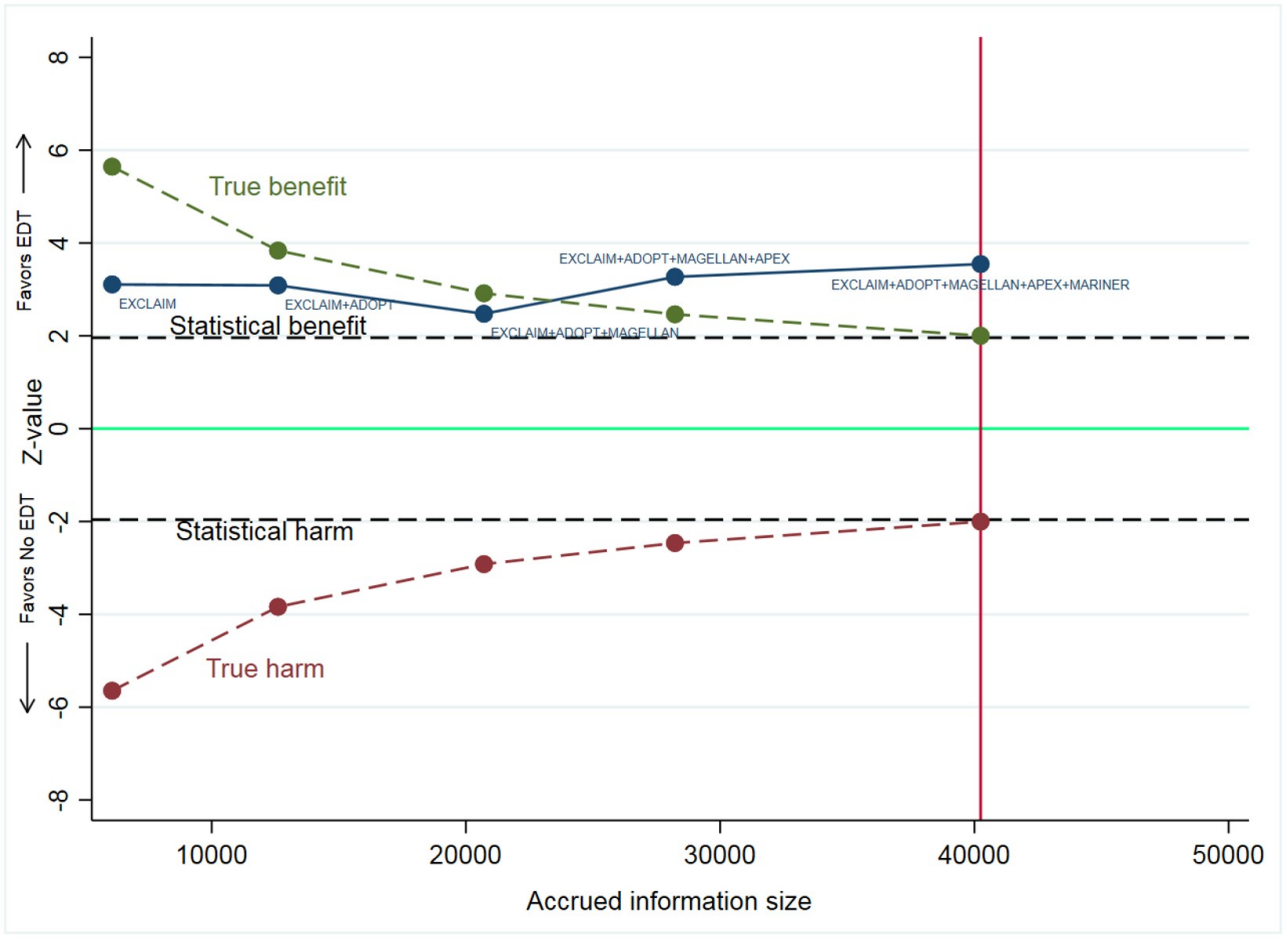
Trial sequential analysis of EDT versus standard-duration thromboprophylaxis in hospitalized medically ill patients for primary efficacy events. The green dashed line represents Lan–DeMets trial sequential boundary for benefit and the maroon dashed line represents Lan–DeMets trial sequential boundary for harm. The blue solid line is cumulative Z-curve derived from random effects cumulative meta-analysis. The solid bright green line indicates line of no difference, and dashed black lines are upper and lower bounds for 95% CI. The total accrued information size for this analysis was 40,247 patients. The cumulative Z-curve surpassed both the dashed black line (line of statistical significance at Z-score of +1.96) and green dashed line (Lan–DeMets trial sequential boundary for benefit), indicating true benefit EDT over standard of care in reducing postdischarge VTE. ADOPT, Apixaban Dosing to Optimize Protection from Thrombosis; APEX, Acute Medically Ill Venous Prevention with Extended Duration Betrixaban; CI, confidence interval; EDT, extended-duration thromboprophylaxis; EXCLAIM, Extended Prophylaxis for Venous ThromboEmbolism in Acutely Ill Medical Patients With Prolonged Immobilization; MAGELLAN, Multicenter, Randomized, Parallel Group Efficacy and Safety Study for the Prevention of Venous Thromboembolism in Hospitalized Acutely Ill Medical Patients Comparing Rivaroxaban with Enoxaparin; MARINER, Medically Ill Patient Assessment of Rivaroxaban versus Placebo in Reducing Post-Discharge Venous Thrombo-Embolism Risk; VTE, venous thromboembolism.

As new trial data became available, the cumulative meta-analysis showed increasing precision of treatment effect, with narrowing of 95% CI (Fig 3). The cumulative Z-curve assessing the effect size of EDT compared with standard of care crossed the statistical boundary and the Lan–DeMets boundary for evidence of a true benefit, indicating robustness of pooled results (Fig 4). Similar findings were observed when analyzing the primary efficacy endpoint selected by each trial.

#### Safety endpoints

**Fig 5:**
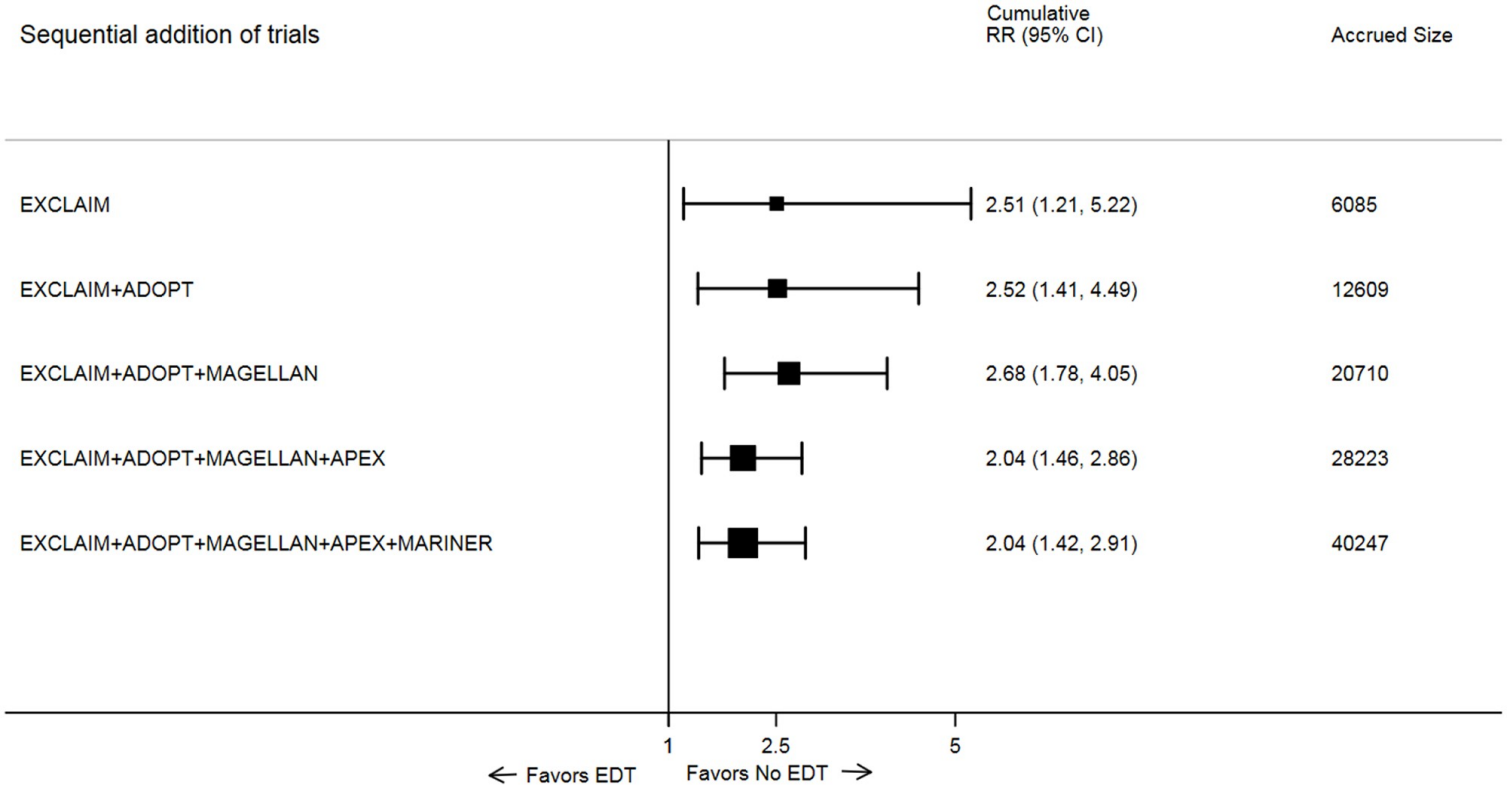
Forest plot for cumulative meta-analysis of EDT versus standard-duration thromboprophylaxis in hospitalized medically ill patients for major or fatal bleeding. Black solid square markers and associated solid lines represent cumulative summary RR and 95% CI after addition of each trial as listed in left column. The size of black markers is proportional to total accrued size. The numerical estimates in the right columns are cumulative RRs and 95% CI after sequentially adding each trial and cumulative sample size after addition of each trial listed in left column. ADOPT, Apixaban Dosing to Optimize Protection from Thrombosis; APEX, Acute Medically Ill Venous Prevention with Extended Duration Betrixaban; CI, confidence interval; EDT, extended-duration thromboprophylaxis; EXCLAIM, Extended Prophylaxis for Venous ThromboEmbolism in Acutely Ill Medical Patients With Prolonged Immobilization; MAGELLAN, Multicenter, Randomized, Parallel Group Efficacy and Safety Study for the Prevention of Venous Thromboembolism in Hospitalized Acutely Ill Medical Patients Comparing Rivaroxaban with Enoxaparin; MARINER, Medically Ill Patient Assessment of Rivaroxaban versus Placebo in Reducing Post-Discharge Venous Thrombo-Embolism Risk; RR, risk ratio.

EDT also significantly increased risk of ISTH-criteria–based major or fatal bleeding (0.6% versus 0.3%; RR: 2.04, 95% CI: 1.42–2.91; *p* < 0.001) in both cumulative meta-analysis and trial sequential analysis (Figs 2 and 5). However, EDT did not significantly increase all-cause mortality (3.3% versus 3.4%; RR: 0.97, 95% CI: 0.87–1.08; *p* = 0.598) as compared with standard of care in both cumulative meta-analysis and trial sequential analysis. The heterogeneity estimates for all safety outcomes are presented in S4 Table.

#### Efficacy and safety of EDT

**Fig 6:**
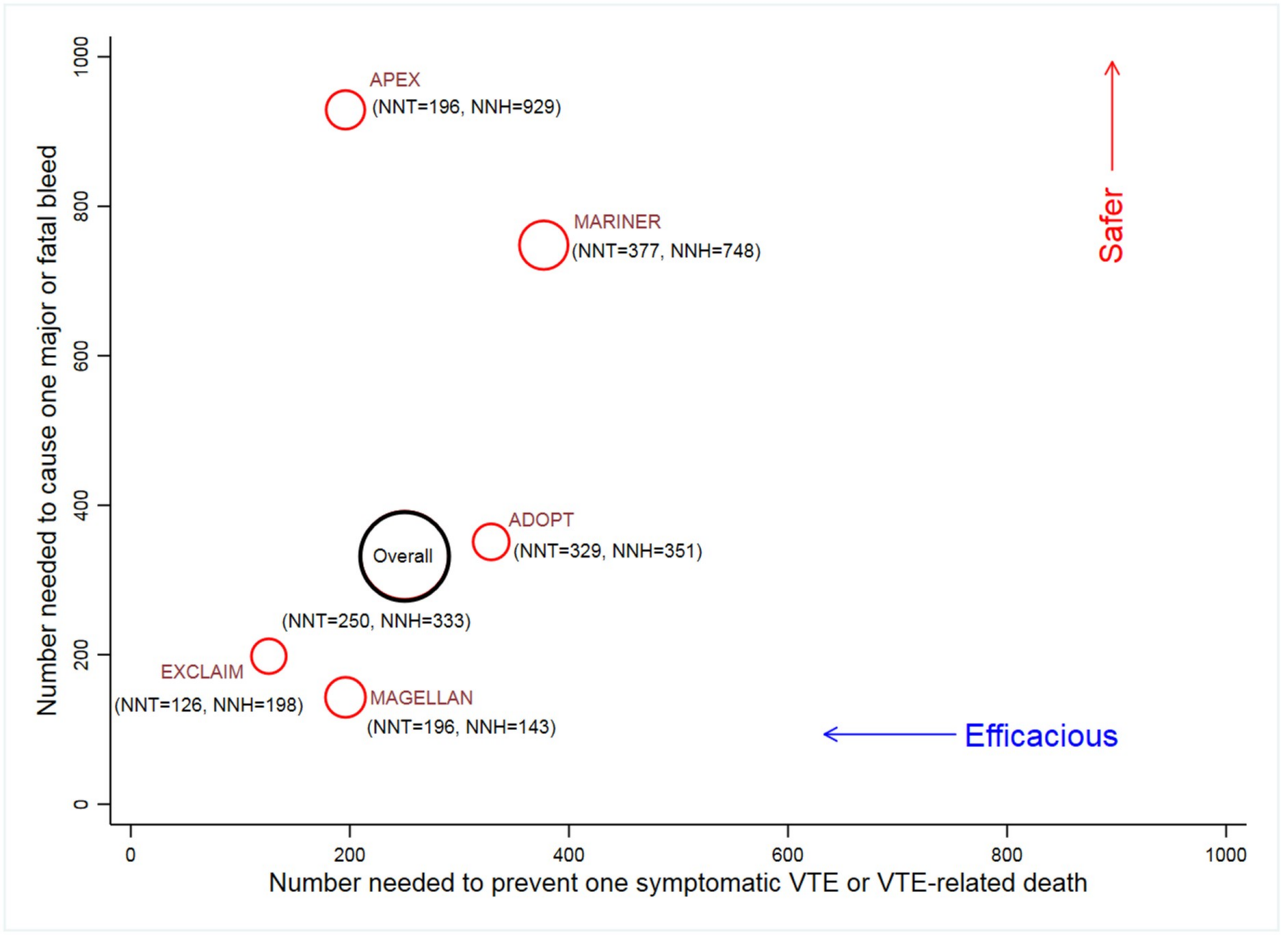
NNT to prevent one symptomatic VTE or VTE-related death and NNH to cause one major or fatal bleeding event for individual trials and overall pooled estimates. The size of red bubbles is proportional to number of patients in the trial, and the size of the black bubble is proportional to accrued information size. The overall pooled estimates for NNH and NNT were calculated by conducting random effects meta-analyses for risk difference for primary efficacy and safety events. Safer implies higher NNH for causing one major or fatal bleeding event. Efficacious implies lower NNT to prevent one symptomatic VTE or VTE-related death. ADOPT, Apixaban Dosing to Optimize Protection from Thrombosis; APEX, Acute Medically Ill Venous Prevention with Extended Duration Betrixaban; EXCLAIM, Extended Prophylaxis for Venous ThromboEmbolism in Acutely Ill Medical Patients With Prolonged Immobilization; MAGELLAN, Multicenter, Randomized, Parallel Group Efficacy and Safety Study for the Prevention of Venous Thromboembolism in Hospitalized Acutely Ill Medical Patients Comparing Rivaroxaban with Enoxaparin; MARINER, Medically Ill Patient Assessment of Rivaroxaban versus Placebo in Reducing Post-Discharge Venous Thrombo-Embolism Risk; NNH, number need to harm; NNT, number needed to treat; VTE, venous thromboembolism.

Using random effects meta-analyses of risk differences, we estimate that the pooled NNT to prevent one symptomatic VTE or VTE-related death was 250 (95% CI: 167–500), whereas pooled NNH to cause one major or fatal bleeding event was 333 (95% CI: 200–1,000) (Fig 6). When trial-specified definitions of primary efficacy endpoints were employed, the pooled NNT to prevent one trial-defined primary efficacy endpoint was 111 (95% CI: 55–333). Fig 6 shows that the Acute Medically Ill Venous Prevention with Extended Duration Betrixaban (APEX) trial had one of the lowest NNTs to prevent symptomatic VTE or VTE-related death and highest NNHs to cause one major or fatal bleed, indicating that EDT among APEX patients was both efficacious and safe as compared with other trials.

## Discussion

In this study, we observed that EDT reduced the risk of symptomatic or fatal VTE when compared with standard-duration thromboprophylaxis (which typically ceases at the time of discharge) among medically ill patients after hospitalization. The observed benefits across trials were directionally consistent and of a similar magnitude and less likely to be due to type I or type II error because cumulative treatment effect from our analyses crossed both statistical and trial sequential boundaries of benefit. The benefits accrued with VTE prevention were, however, counterbalanced by an increased rate of major or fatal bleeding with EDT. The overall risks and benefits observed across the 5 trials were modest, as evidenced by high summary NNT and NNH.

Patients discharged from hospital after medical illness face an ongoing risk for development of VTE for up to 6 weeks. These thrombotic events that develop within the postdischarge vulnerable period confer clinical morbidity, mortality, and excess healthcare expenditure because of the high rate of short-term readmission [21]. Five clinical trials, including the recently published MARINER study [5], have evaluated a strategy of EDT using either a low-molecular weight heparin or a factor Xa inhibitor in an effort to abrogate this heightened postdischarge risk for VTE. Individual trials have contributed to uncertainty about the overall clinical utility of EDT, either by not meeting their primary efficacy endpoints or by demonstrating excess harm from bleeding risks. Moreover, when each trial is examined using the fragility index—which measures the number of additional events needed in the control arm to create a null effect—the statistical robustness of individual RCTs of EDT was low such that the fragility index ranges from as low as 6 to as many as 16 for “positive” trials. Indeed, these fragility indices for individual studies are lower than those reported for trials that are ultimately used to develop and support guideline recommendations [22]. Thus, a meta-analysis performed using a clinically relevant efficacy endpoint can provide a more durable metric for reliance in the robustness of the finding of benefit.

Clinical interpretation of these aggregate findings must account for variation in the definitions of trial enrollment criteria, individual trial definitions of primary efficacy and safety endpoints, and EDT approaches in each trial using different agents with distinct pharmacological profiles and doses. The typical patient enrolled in these trials was relatively similar across trials—older, relatively immobile, with acute medical illness. However, some important differences deserve discussion. In 3 trials, certain risk-enrichment strategies were applied based on clinical parameters [2], age, and elevated biomarkers such as D-dimer [4] or the use of a validated clinical risk score and D-dimer [5]. All trials enrolled a broad range of hospitalized patients but varied in the proportions of included patients with specific medical conditions. For instance, the proportion of enrolled patients who were hospitalized for heart failure ranged from 18% [9] to 45% [4]. Finally, the EDT strategy employed was different across trials even for the 2 studies evaluating the same agent, rivaroxaban. While MAGELLAN (Multicenter, Randomized, Parallel Group Efficacy and Safety Study for the Prevention of Venous Thromboembolism in Hospitalized Acutely Ill Medical Patients Comparing Rivaroxaban with Enoxaparin) [3] evaluated fixed-dose rivaroxaban beginning within 72 hours of hospitalization, MARINER [5] initiated rivaroxaban at the time of discharge and used dose-reduced rivaroxaban in patients with moderate renal insufficiency [5]. Most trials included asymptomatic DVT, as ascertained by routine surveillance ultrasonography, as part of the primary efficacy endpoint. We focused our analysis on clinically relevant, symptomatic VTE events or VTE-related deaths (the primary endpoint of the MARINER trial [5]), as opposed to asymptomatic VTE events detected on protocolized venous ultrasonography.

The results of this meta-analysis provide a more comprehensive understanding of the use of EDT as a therapeutic strategy. For instance, the US Food and Drug Association recently approved the factor Xa inhibitor betrixaban based on exploratory analyses of aggregate data from the APEX trial despite it not having met its prespecified primary efficacy endpoint [4]. The recent MARINER trial [5], which studied rivaroxaban, did not meet its primary endpoint, although some secondary efficacy endpoints were improved by EDT. Such disparate outcomes for efficacy, added to relatively consistent safety signals for increases in major bleeding across these various trials, may lead to the development of clinical uncertainty and difficulty in implementation of trial data. Thus, we employed the meta-analytic approach to allow for greater clarity in understanding the efficacy signals for EDT irrespective of the therapeutic agent employed, determine the overall robustness of these findings, and allow for the assessment of the relative balance of benefit and harm. This could facilitate the development of consensus or guideline statements to provide sufficient precision in direction for clinicians.

Nonetheless, our review is not without limitations. Amalgamation of data in the form of meta-analyses has well-recognized limitations [20]. The included studies differed in the type of drugs, dosage, duration of treatment, patient population, and DVT detection protocols included. Although the treatment effects were similar across trials, we observed evidence of statistical heterogeneity across trials, and therefore the impact of variation in patient profiles and treatment protocols on the summary treatment effects cannot be excluded. The findings of these analyses should not replace the clinical judgement of a treating physician but might help them make personalized decisions based on risk assessment of VTE and bleeding. This remains a topic of discussion, and we hope that future studies will help to derive the optimal protocols to address this personalized risk–benefit calculus.

In summary, our meta-analysis demonstrated that use of a post-hospital discharge EDT strategy for a 4-to-6-week period reduced symptomatic or fatal VTE events. These modest benefits were observed at the expense of increased risk of major or fatal bleeding events. Given the relatively infrequent occurrence of these events, we estimate that 250 patients would need to be treated with EDT to prevent one symptomatic or fatal VTE event, and 333 patients would need to be exposed to EDT to cause one major or fatal bleeding event. Further investigations are required to define risks and benefits within specific and discrete populations of medically ill patients, evaluate the cost-effectiveness of EDT, and develop pathways for targeted implementation of this postdischarge strategy in appropriately selected patients.

## Supporting information

S1 PRISMA checklist PRISMA, Preferred Reporting Items for Systematic Reviews and Meta-Analyses. (DOCX)

S1 Search Strategy (DOCX)

S1 Table Definitions of symptomatic VTE/VTE-related death across trials. VTE, venous thromboembolism.
(DOCX)

S2 Table Definitions of major bleeding across trials. (DOCX)

S3 Table Measures of heterogeneity. (DOCX)

S4 Table Number of events (*n*) and denominators (*N*) across included studies. (DOCX)

## Abbreviations

ADOPT: Apixaban Dosing to Optimize Protection from Thrombosis
APEX: Acute Medically Ill Venous Prevention with Extended Duration Betrixaban
CI: confidence interval
CrCl: creatinine clearance
DVT: deep vein thrombosis
EDT: extended-duration thromboprophylaxis
EXCLAIM: Extended Prophylaxis for Venous ThromboEmbolism in Acutely Ill Medical Patients With Prolonged Immobilization
ISTH: International Society on Thrombosis and Haemostasis
MAGELLAN: Multicenter, Randomized, Parallel Group Efficacy and Safety Study for the Prevention of Venous Thromboembolism in Hospitalized Acutely Ill Medical Patients Comparing Rivaroxaban with Enoxaparin
MARINER: Medically Ill Patient Assessment of Rivaroxaban versus Placebo in Reducing Post-Discharge Venous Thrombo-Embolism Risk
MeSH: Medical Subject Headings
NNH: number needed to harm
NNT: number needed to treat
NR: not recorded
NYHA: New York Heart Association
PRISMA: Preferred Reporting Items for Systematic Reviews and Meta-Analyses
RCT: randomized clinical trial
RoB: risk of bias
RR: risk ratio
SD: standard deviation
SDT: standard-duration thrombosis
VTE: venous thromboembolism

## References

[1] Kahn SR, Lim W, Dunn AS, Cushman M, Dentali F, Akl EA, et al Prevention of VTE in Nonsurgical Patients: Antithrombotic Therapy and Prevention of Thrombosis, 9th ed: American College of Chest Physicians Evidence-Based Clinical Practice Guidelines. Chest. 2012;141(2 Suppl):e195S–226S. 10.1378/chest.11-2296 22315261 PMC3278052

[2] Goldhaber SZ, Leizorovicz A, Kakkar AK, Haas SK, Merli G, Knabb RM, et al Apixaban versus enoxaparin for thromboprophylaxis in medically ill patients. N Engl J Med. 2011;365(23):2167–77. 10.1056/NEJMoa1110899 22077144

[3] Cohen AT, Spiro TE, Spyropoulos AC, Committee MS. Rivaroxaban for thromboprophylaxis in acutely ill medical patients. N Engl J Med. 2013;368(20):1945–6. 10.1056/NEJMc1303641 23675665

[4] Cohen AT, Harrington RA, Goldhaber SZ, Hull RD, Wiens BL, Gold A, et al Extended Thromboprophylaxis with Betrixaban in Acutely Ill Medical Patients. The New England journal of medicine. 2016;375(6):534–44. 10.1056/NEJMoa1601747 27232649

[5] Spyropoulos AC, Ageno W, Albers GW, Elliott CG, Halperin JL, Hiatt WR, et al Rivaroxaban for Thromboprophylaxis after Hospitalization for Medical Illness. New England Journal of Medicine.2018;379(12):1118–27. 10.1056/NEJMoa1805090 30145946

[6] Liew AY, Piran S, Eikelboom JW, Douketis JD. Extended-duration versus short-duration pharmacological thromboprophylaxis in acutely Ill hospitalized medical patients: a systematic review and meta-analysis of randomized controlled trials. J Thromb Thrombolysis. 2017;43(3):291–301. 10.1007/s11239-016-1461-1 27900627

[7] Tao DL, Bien JY, DeLoughery TG, Shatzel JJ. Extended thromboprophylaxis with direct oral anticoagulants for medical patients: a systematic review and meta-analysis. Blood. 2017;129(5):653–5. 10.1182/blood-2016-10-747931 27998890

[8] Dentali F, Mumoli N, Prisco D, Fontanella A, Di Minno MN. Efficacy and safety of extended thromboprophylaxis for medically ill patients. A meta-analysis of randomised controlled trials. Thromb Haemost. 2017;117(3):606–17. 10.1160/TH16-08-0595 28078350

[9] Hull RD, Schellong SM, Tapson VF, Monreal M, Samama MM, Nicol P, et al Extended-duration venous thromboembolism prophylaxis in acutely ill medical patients with recently reduced mobility: a randomized trial. Annals of internal medicine. 2010;153(1):8–18. 10.7326/0003-4819-153-1-201007060-00004 20621900

[10] Krist AH. "Needs More Research"-Implications of the Proteus Effect for Researchers and Evidence Adopters. Mayo Clinic Proceedings. 2018;93(3):273–5. 10.1016/j.mayocp.2018.01.013 29477780

[11] Pfeiffer T, Bertram L, Ioannidis JP. Quantifying selective reporting and the Proteus phenomenon for multiple datasets with similar bias. PLoS ONE. 2011;6(3):e18362 10.1371/journal.pone.0018362 21479240 PMC3066227

[12] Schulman S, Kearon C. Definition of major bleeding in clinical investigations of antihemostatic medicinal products in non-surgical patients. Journal of thrombosis and haemostasis: JTH. 2005;3(4):692–4. 10.1111/j.1538-7836.2005.01204.x 15842354

[13] Higgins JP, Thompson SG. Quantifying heterogeneity in a meta-analysis. Stat Med. 2002;21(11):1539–58. 10.1002/sim.1186 12111919

[14] Egger M, Davey Smith G, Schneider M, Minder C. Bias in meta-analysis detected by a simple, graphical test. Bmj. 1997;315(7109):629–34. 9310563 10.1136/bmj.315.7109.629 PMC2127453

[15] Duval S, Tweedie R. Trim and fill: A simple funnel-plot-based method of testing and adjusting for publication bias in meta-analysis. Biometrics. 2000;56(2):455–63. 10877304 10.1111/j.0006-341x.2000.00455.x

[16] Lau J, Antman EM, Jimenez-Silva J, Kupelnick B, Mosteller F, Chalmers TC. Cumulative meta-analysis of therapeutic trials for myocardial infarction. N Engl J Med. 1992;327(4):248–54. 10.1056/NEJM199207233270406 1614465

[17] Wetterslev J, Jakobsen JC, Gluud C. Trial Sequential Analysis in systematic reviews with meta-analysis. BMC medical research methodology. 2017;17(1):39 10.1186/s12874-017-0315-7 28264661 PMC5397700

[18] Higgins JPT S J, Savović J, Page MJ, Hróbjartsson A, Boutron I, Reeves B, Eldridge S. A revised tool for assessing risk of bias in randomized trials. In: Chandler J., McKenzie J., Boutron I., Welch V. (editors). Cochrane Methods Cochrane Database of Systematic Reviews. 2016;10(Suppl 1):29–31.

[19] Miladinovic BHI, Djulbegovic B. Trial sequential boundaries for cumulative meta-analyses. Stata Journal 2013;13(1):77–91.

[20] Kalra R, Arora P, Morgan C, Hage FG, Iskandrian AE, Bajaj NS. Conducting and interpreting high-quality systematic reviews and meta-analyses. J Nucl Cardiol. 2017;24(2):471–81. 10.1007/s12350-016-0598-9 27484213

[21] Spyropoulos AC, Lin J. Direct medical costs of venous thromboembolism and subsequent hospital readmission rates: an administrative claims analysis from 30 managed care organizations. Journal of managed care pharmacy: JMCP. 2007;13(6):475–86. 10.18553/jmcp.2007.13.6.475 17672809 PMC10437642

[22] Docherty KF, Campbell RT, Jhund PS, Petrie MC, McMurray JJV. How robust are clinical trials in heart failure? Eur Heart J. 2017;38(5):338–45. 10.1093/eurheartj/ehw427 27742808

