# Supplementary material for: Extended prophylaxis for venous thromboembolism after hospitalization for medical illness: A trial sequential and cumulative meta-analysis
Source: PLoS Med. 2019 Apr 29;16(4):e1002797. doi: 10.1371/journal.pmed.1002797 (PMC6488047; doi:10.1371/journal.pmed.1002797)
Supplement: S1 Search Strategy — (DOCX) [file pmed.1002797.s002.docx]

**S2 Search Strategy**

**Pubmed/MEDLINE-n=48**

("Anticoagulants"[Mesh] OR "Heparin"[Mesh] OR "Warfarin"[Mesh] OR "Factor Xa Inhibitors" [Pharmacological Action] OR "Rivaroxaban"[Mesh] OR "Antithrombins" [Pharmacological Action] OR "Dabigatran"[Mesh] OR "Aspirin"[Mesh]) AND ("extended" OR "extended-duration") AND ("Venous Thromboembolism"[Mesh]) – limit to clinical trials and humans

**EMBASE n=148**

((**'anticoagulants'**/exp OR **'anticoagulants'** OR **'heparin'**/exp OR **'heparin'** OR **'enoxaparin'**/exp OR **'enoxaparin'** OR **'warfarin'**/exp OR **'warfarin'** OR **'rivaroxaban'**/exp OR **'rivaroxaban'** OR **'apixaban'**/exp OR **'apixaban'** OR **'edoxaban'**/exp OR **'edoxaban'** OR **'betrixaban'**/exp OR **'betrixaban'** OR **'dabigatran'**/exp OR **'dabigatran'** OR **'aspirin'**/exp OR **'aspirin'**) AND **'extended'** OR **'extended-duration'**) AND (**'venous thromboembolism'**/exp OR **'venous thromboembolism'**) AND ([controlled clinical trial]/lim OR [randomized controlled trial]/lim) AND ([article]/lim OR [article in press]/lim OR [conference abstract]/lim OR [conference paper]/lim OR [conference review]/lim) AND [humans]/lim AND [english]/lim AND ([embase]/lim OR [medline]/lim)

**Cochrane Central n=157 trials**

("Anticoagulants" OR "Heparin" OR “Warfarin” OR "Enoxaparin" OR "Warfarin" OR "Rivaroxaban" OR "Apixaban" OR "Edoxaban" OR "Betrixaban" OR "Dabigatran" OR "Aspirin") AND ("extended" OR "extended-duration") AND ("Venous Thromboembolism")

**ClinicalTrials.gov n=9**

("Anticoagulants" OR "Heparin" OR “Warfarin” OR "Enoxaparin" OR "Warfarin" OR "Rivaroxaban" OR "Apixaban" OR "Edoxaban" OR "Betrixaban" OR "Dabigatran" OR "Aspirin") AND ("extended" OR "extended-duration") AND ("Venous Thromboembolism") – limit to “interventional (clinical trials)” and “completed
