# Supplementary material for: Extended prophylaxis for venous thromboembolism after hospitalization for medical illness: A trial sequential and cumulative meta-analysis
Source: PLoS Med. 2019 Apr 29;16(4):e1002797. doi: 10.1371/journal.pmed.1002797 (PMC6488047; doi:10.1371/journal.pmed.1002797)
Supplement: S1 Table — VTE, venous thromboembolism. (DOCX) [file pmed.1002797.s003.docx]

| **Study** | **Trial-based definition** |
| --- | --- |
| **MARINER (5)** | - **Symptomatic VTE**: symptomatic DVT or nonfatal PE - **VTE-related death**: death due to PE or death in which PE could not be ruled out as cause |
| **APEX (4)** | - **Symptomatic VTE**: death from venous thromboembolism, nonfatal pulmonary embolism, or symptomatic deep-vein thrombosis - **VTE-related death**: death adjudicated as a confirmed, probable, or possible fatal PE/VTE. |
| **MAGELLAN (3)** | - **Symptomatic VTE**: DVT or PE - **VTE-related death**: Not available |
| **ADOPT (2)** | - **Symptomatic VTE**: Not available - **VTE-related death**: sudden death for which pulmonary embolism could not be excluded as a cause |
| **EXCLAIM (9)** | - **Symptomatic VTE**: the composite of symptomatic proximal DVT, symptomatic pulmonary embolism, or fatal pulmonary embolism - **VTE-related death:** Not available |

S1 Table: Definitions of symptomatic venous thromboembolism (VTE)/ VTE-related death across trials

DVT: Deep vein thrombosis; PE: Pulmonary embolism
