# Supplementary material for: Extended prophylaxis for venous thromboembolism after hospitalization for medical illness: A trial sequential and cumulative meta-analysis
Source: PLoS Med. 2019 Apr 29;16(4):e1002797. doi: 10.1371/journal.pmed.1002797 (PMC6488047; doi:10.1371/journal.pmed.1002797)
Supplement: S2 Table — (DOCX) [file pmed.1002797.s004.docx]

| **Study** | **Major bleeding definition** |
| --- | --- |
| **MARINER (5)** | Defined as either of the following (as per ISTH):   - Fall in hemoglobin level of 2 g/dL or more or - A transfusion of two or more units of packed red blood cells, or whole blood, or - Bleeding in a critical site: intracranial, intraspinal, intraocular, pericardial, intraarticular, intramuscular with compartment syndrome, retroperitoneal, or - A fatal outcome |
| **APEX (4)** | Defined as clinically overt bleeding that is associated with either of the following:   - A fall in hemoglobin of 2 g/dL or more, or - A transfusion of 2 or more units of packed red blood cells or whole blood, or - A critical site: intracranial, intraspinal, intraocular, pericardial, intra-articular, intramuscular with compartment syndrome, retroperitoneal, or - Death |
| **MAGELLAN (3)** | Defined as per ISTH as clinically overt bleeding that is associated with either of the following:   - Bleeding associated with a reduction in hemoglobin of at least 2 g/dl or - Leading to a transfusion of at least 2 units of blood or packed cells or - Symptomatic bleeding in a critical area or organ: intraocular,   intracranial, intraspinal, or intramuscular with compartment syndrome, retroperitoneal bleeding, intra-articular bleeding or pericardial bleeding or   - A fatal outcome |
| **ADOPT (2)** | Defined as per ISTH as clinically overt bleeding that is associated with either of the following:   - A decrease in hemoglobin of 2 g/dL or more over a 24-hour period - A transfusion of 2 or more units of packed red blood cells - Bleeding that occurs in at least one of the following critical sites: Intracranial; Intra-spinal; Intraocular (within the corpus of the eye; thus, a conjunctival bleed is not an intraocular bleed); Pericardial; An operated joint and requires re-operation or intervention; Intramuscular with compartment syndrome; Retroperitoneal; - Bleeding that is fatal |
| **EXCLAIM (9)** | Major hemorrhages defined as either of the following:   - Overt and associated with death or - a decrease in hemoglobin level of at least 20 g/L or - A transfusion of at least 2 units of packed red blood cells or whole blood; surgical intervention or - Retroperitoneal, intracranial, or intraocular bleeding. |

S2 Table: Definitions of major bleeding across trials

ISTH: International Society on Thrombosis and Haemostasis
