# Supplementary material for: Extended prophylaxis for venous thromboembolism after hospitalization for medical illness: A trial sequential and cumulative meta-analysis
Source: PLoS Med. 2019 Apr 29;16(4):e1002797. doi: 10.1371/journal.pmed.1002797 (PMC6488047; doi:10.1371/journal.pmed.1002797)
Supplement: S3 Table — (DOCX) [file pmed.1002797.s005.docx]

| Outcome | I^2^ (95% CI) | Cochrane Q, df, p-value | H (95% CI) | **τ^2^** | |
| --- | --- | --- | --- | --- | --- |
|  |  |  |  | ML | REML |
| Symptomatic VTE or VTE- related death (Primary efficacy outcome) | 47% (0%-81%) | 7.59,4, 0.110 | 1.4 (1-2.3) | 0 | 0 |
| Major or Fatal Bleed | 23% (0%-68%) | 5.18,4, 0.269 | 1.1 (1-1.8) | 0.030 | 0.058 |
| All-cause mortality | 0% (0%-7%) | 3.13,4,  0.678 | 1 (1.0-2.2) | 0 | 0 |

**S3** Supplemental Table: Measures of heterogeneity.

df: degrees of freedom; ML: Maximal likelihood; REML: Restricted Maximal likelihood; VTE = venous thromboembolism
