# Supplementary material for: Extended prophylaxis for venous thromboembolism after hospitalization for medical illness: A trial sequential and cumulative meta-analysis
Source: PLoS Med. 2019 Apr 29;16(4):e1002797. doi: 10.1371/journal.pmed.1002797 (PMC6488047; doi:10.1371/journal.pmed.1002797)
Supplement: S4 Table — (DOCX) [file pmed.1002797.s006.docx]

| Study | Study Arm | Symptomatic VTE or VTE- related death | Trial-defined efficacy outcome | Major or fatal Bleed | All-cause mortality |
| --- | --- | --- | --- | --- | --- |
| MARINER (5) | Intervention Arm (n/N) | 50/6007 | 50/6007 | 17/5982 | 71/6007 |
|  | Control Arm (n/N) | 66/6012 | 66/6012 | 9/5980 | 89/6012 |
| APEX (4) | Intervention Arm (n/N) | 35/3721 | 165/3112 | 25/3716 | 210/3716 |
|  | Control Arm (n/N) | 54/3720 | 223/3174 | 21/3716 | 215/3716 |
| MAGELLAN (3) | Intervention Arm (n/N) | 42/2967 | 131/2967 | 43/3997 | 159/3096 |
|  | Control Arm (n/N) | 59/3057 | 175/3057 | 15/4001 | 153/3169 |
| ADOPT (2) | Intervention Arm (n/N) | 8/3255 | 60/2211 | 15/3184 | 131/3255 |
|  | Control Arm (n/N) | 18/3273 | 70/2284 | 6/3217 | 133/3273 |
| EXCLAIM (9) | Intervention Arm (n/N) | 5/2485 | 61/2485 | 25/2975 | 60/2975 |
|  | Control Arm (n/N) | 25/2510 | 100/2510 | 10/2988 | 65/2988 |

**S4** Supplemental Table: Number of events (n) and denominators (N) across included studies
